# Paradoxical effects of cigarette smoke and COPD on SARS-CoV-2 infection and disease

**DOI:** 10.1186/s12890-021-01639-8

**Published:** 2021-08-23

**Authors:** M. Tomchaney, M. Contoli, J. Mayo, S. Baraldo, S. Li, C. R. Cabel, D. A. Bull, S. Lick, J. Malo, S. Knoper, S. S. Kim, J. Tram, J. Rojas-Quintero, M. Kraft, J. G. Ledford, Y. Tesfaigzi, F. D. Martinez, C. A. Thorne, F. Kheradmand, S. K. Campos, A. Papi, F. Polverino

**Affiliations:** 1 Asthma and Airway Disease Research Center, University of Arizona, Tucson, AZ 85719 USA; 2 Respiratory Unit, Department of Translational Medicine, University of Ferrara, Ferrara, Italy; 3 Department of Cardiological, Thoracic, Vascular Sciences and Public Health, University of Padova, Padova, Italy; 4 Department of Immunobiology, University of Arizona College of Medicine, Tucson, USA; 5 Thoracic Surgery, University of Arizona, Tucson, USA; 6 Thoracic Surgery, Northwester University, Chicago, IL USA; 7 Brigham and Women’s Hospital, Harvard Medical School, Boston, MA USA; 8 Department of Cellular and Molecular Medicine, University of Arizona Cancer Center, Tucson, USA; 9 BIO5 Institute, University of Arizona, Tucson, USA; 10 Baylor College of Medicine, Houston, TX USA

## Abstract

**Background:**

How cigarette smoke (CS) and chronic obstructive pulmonary disease (COPD) affect severe acute respiratory syndrome coronavirus 2 (SARS-CoV2) infection and severity is controversial. We investigated the effects of COPD and CS on the expression of SARS-CoV-2 entry receptor ACE2 in vivo in COPD patients and controls and in CS-exposed mice, and the effects of CS on SARS-CoV-2 infection in human bronchial epithelial cells in vitro.

**Methods:**

We quantified: (1) pulmonary ACE2 protein levels by immunostaining and ELISA, and both ACE2 and/or TMPRSS2 mRNA levels by RT-qPCR in two independent human cohorts; and (2) pulmonary ACE2 protein levels by immunostaining and ELISA in C57BL/6 WT mice exposed to air or CS for up to 6 months. The effects of CS exposure on SARS-CoV-2 infection were evaluated after in vitro infection of Calu-3 cells and differentiated human bronchial epithelial cells (HBECs), respectively.

**Results:**

ACE2 protein and mRNA levels were decreased in peripheral airways from COPD patients versus controls but similar in central airways. Mice exposed to CS had decreased ACE2 protein levels in their bronchial and alveolar epithelia versus air-exposed mice. CS treatment decreased viral replication in Calu-3 cells, as determined by immunofluorescence staining for replicative double-stranded RNA (dsRNA) and western blot for viral N protein. Acute CS exposure decreased in vitro SARS-CoV-2 replication in HBECs, as determined by plaque assay and RT-qPCR.

**Conclusions:**

ACE2 levels were decreased in both bronchial and alveolar epithelial cells from COPD patients versus controls, and from CS-exposed versus air-exposed mice. CS-pre-exposure potently inhibited SARS-CoV-2 replication in vitro. These findings urge to investigate further the controversial effects of CS and COPD on SARS-CoV-2 infection.

**Supplementary Information:**

The online version contains supplementary material available at 10.1186/s12890-021-01639-8.

## Background

Coronavirus disease-19 (COVID-19), is an acute and heterogeneous clinical condition caused by the newly identified severe acute respiratory syndrome Coronavirus 2 (SARS-CoV2) [1]. In most cases, the disease is characterized by a mild presentation of respiratory symptoms and/or in a variable proportion gastrointestinal, neurologic, and cutaneous symptoms [2]. When severe, the disease can lead to lethal acute respiratory distress syndrome. Despite the tremendous worldwide burden, our understanding of the pathophysiology and treatment of SARS-CoV-2 illnesses is still limited.

The elderly and patients with comorbidities (mainly cardio/respiratory diseases, diabetes, and kidney failure) are at the highest risk of poor prognosis and death from COVID-19 [3]. COPD is one of the most frequent non-communicable chronic conditions affecting millions worldwide [4]. COPD patients are mainly elderly and with frequent comorbid conditions. Because of deficient immune responses [5], COPD is associated with increased susceptibility to infections, and viral infections are one of the main causes of acute exacerbations of COPD. Therefore, the SARS-CoV-2 pandemic could have a dramatic clinical impact in COPD patients [6]. Interestingly, the epidemiological data published so far show a lower than expected prevalence of active smokers and COPD among COVID-19 patients [7–14]. On the other hand, following SARS-CoV-2 infection, COPD patients are at high risk of poor outcomes, including intensive care unit admission and death [15].

SARS-CoV-2 uses the angiotensin-converting enzyme 2 (ACE2) as the host cell entry receptor [16]. The binding of SARS-CoV-2 spike (S) protein to ACE2 triggers a complex series of biochemical (i.e., activation of specific proteases including Transmembrane Serine Protease 2—TMPRSS2) and molecular signals leading to internalization of the virus. ACE2 is expressed in the lung, and its expression declines from the conducting to the respiratory airways, waning in the more distal bronchiolar and alveolar lung regions [17]. There have been mixed reports on the effect of cigarette smoke (CS) and COPD on the expression of ACE2, with the great majority of data showing an up-regulation of ACE2 in the lungs of smokers and COPD patients [18–21], and some other data showing that nicotine downregulates the ACE2 axis [22–24]. Thus, the impact of CS and COPD on SARS-CoV-2 infection is still a matter of debate [25].

In this study, we evaluated: (1) ACE2 and TMPRSS2 expression in the airways of smokers with COPD, compared to smoker and never smoker (NS) controls, and (2) ACE2 expression in lungs of mice acutely and chronically exposed to CS; and (3) the effects of CS extract (CSE) or direct CS exposure on SARS-CoV-2 in vitro infection in Calu3 lung adenocarcinoma epithelial cells and differentiated primary human bronchial epithelium.

## Methods

### Study population

The study conformed to the Declaration of Helsinki, and the work was approved by the institutional ethic committees (University of Arizona IRB #1811124026 and University of Ferrara IRB #080399). Informed written consent was obtained from each subject.

Lung specimens were obtained from different cohorts of COPD patients (according to Global Initiative for Chronic Obstructive Lung Disease definition), smokers without COPD and control non-smokers (NS) as follows. Cohort 1: smokers with pulmonary nodules who had lung resection at Ferrara University Hospital in Italy [26]; Cohort 2: patients who underwent surgery for lung nodules or lung transplant at the University of Arizona [27]. Explanted lungs from NS who died of extrapulmonary causes from the Arizona Donor Network (both from Tucson, AZ) were used as control lungs.

All the subjects defined as “smokers” had a smoking history of at least 10 pack/years, and all the subjects defined as former smokers had quitted smoking at least 1 year prior to the study.

All the ever smokers with and without COPD underwent spirometry according to international guidelines. Patients with COPD were defined according to the Global initiative for Obstructive Lung Disease recommendations. The exclusion criteria were evidence of respiratory tract infection at the time of lung tissue sampling, presence of concomitant chronic lung disease or metastatic cancer, autoimmune disease, immunosuppressive therapy or chemotherapy. In both cohorts, excluding the COPD transplants, the lung tissue was obtained at a maximum distance from the pulmonary lesions and without signs of retro-obstructive pneumonia or tumor invasion.

The control and COPD patients recruited at the University of Arizona (USA) were significantly younger compared to patients recruited at the University of Ferrara, Italy. All the groups of patients recruited at the University of Arizona (USA) had significantly lower lung function compared to patients recruited at the University of Ferrara, Italy. A higher but not significant proportion of COPD patients in the cohort of COPD patients recruited at the University of Arizona (USA) were on ICS containing pharmacological regimens compared to patients recruited at the University of Ferrara, Italy.

The study conformed to the Declaration of Helsinki, the work was approved by the institutional local ethic committees, and informed written consent was obtained from each subject.

### Detection of ACE2 by immunohistochemistry in central and peripheral airways

Formalin-fixed paraffin embedded (FFPE) human peripheral lung tissues and human bronchial rings were collected at the University of Ferrara, Italy. After deparaffinization and rehydration, the sections to be stained, immersed in retrieval solution citrate pH 6.0, were incubated in a microwave oven on high power for 40 min to expose the immunoreactive epitopes. Then, after a 30 min cooling, endogenous peroxidase activity was blocked by incubating slides for 20 min in 3% hydrogen peroxide (H_2_O_2_), followed by washing in tap water. Non-specific labeling was blocked by coating with blocking serum (5% normal goat serum (Thermofisher Scientific) in PBS; cell membranes were permeabilized adding 0.1% saponin to the PBS) for 20 min at room temperature. After removing the blocking serum, the sections were immediately incubated with a primary antibody (rabbit polyclonal anti-ACE2; PA5-20046, Invitrogen) at the dilution of 0.005 μg/μl (1:200) in PBS/0.1% saponin for 60 min at room temperature. After repeated washing steps with PBS/0.1% saponin, the sections were subsequently incubated with goat anti-rabbit biotinylated antibody (BA-1000, Vector Laboratories) diluted in PBS/0.1% saponin for 30 min at room temperature. After further washing with PBS/0.1% saponin, the sections were subsequently incubated with ABC reagent (Vector ABC Kit; PK-6100, Vector Laboratories) diluted in PBS for 30 min at room temperature. After more washings with PBS/0.1% saponin, slides were incubated with chromogen-fast diaminobenzidine (DAB) as chromogenic substance (D4293-50SET, Sigma) for 2 min. Finally, sections were counterstained in hematoxylin QS (H-3404, Vector Laboratories), dehydrated, cleared and mounted with a cover glass on permanent mounting medium (VectaMount; H-5000, Vector Laboratories). Kidney tissue samples were utilized as positive controls, while negative controls were obtained either omitting the primary antibody or using isotype IgG, and revealed no signal. Cell-type specific staining was visualized under a light microscope (LeicaDM2000) connected to a video recorder and a computerized image analysis system (Leica LAS w3.8). ACE2 positive staining in epithelial cells was quantified in both central and peripheral airways and was expressed as the percentage of positively stained epithelial area over the total area of the epithelium.

### Detection of ACE2 by immunofluorescence in peripheral airways and alveolar epithelial cells

Lung FFPE sections were deparaffinized, and antigen (Ag) retrieval was performed by treating the slides immersed in 0.01 M sodium citrate and 2 mM citrate buffer (pH 6.0) in a microwave. To identify ACE2-positive within the alveolar and bronchial epithelium and in blood vessels, lung sections were triple-immunostained with: (1) murine anti-ACE2 IgG (1:20 overnight a 4 °C, Santa-Cruz biotechnology, Dallas, TX, sc-390851) followed by Alexa-488 IgG (1:100 for 1 h at 37 °C, Fisher Scientific, Hampton, NH, A11001); and 2) rabbit anti Pan-Cytokeratin (as a marker of epithelial cells, 1:50 overnight a 4 °C, Bioss Antibodies, Woburn, MA, bs-1712R) followed by to Alexa-568 IgG (1:100 for 1 h at 37 °C, Fisher Scientific, Hampton, NH, A11036); and 3) sheep anti-VWF IgG (as a marker of endothelial cells, 1:50 for 2 h at 37 °C, Abcam, Cambridge, UK, ab11713) followed by Alexa-647 IgG (1:100 for 1 h at 37 °C, Abcam, Cambridge, UK, ab150179).

To identify ACE2-positive goblet cells within the bronchial epithelium, lung sections were double immunostained with: (1) mouse anti-ACE2 IgG (1:20 overnight a 4 °C, Santa-Cruz biotechnology, sc-390851) followed by Alexa-568 IgG (1:100 for 1 h at 37 °C Fisher Scientific, Hampton, NH, A11004); and (2) rabbit MUC5AC (1:50 overnight a 4 °C, Bioss Antibodies, Woburn, MA bs-7166R) followed by Alexa-488 IgG (1:100 for 1 h at 37 °C, Fisher Scientific, A11034). Lung sections were also immuno-stained with appropriate isotype-matched non-immune control antibodies. For each sample, 20 randomly-selected high-magnification fields were evaluated using an Olympus epi-fluorescence microscope (Olympus Global Corporation). The number of parenchymal, vascular, and bronchial ACE2+ cells were counted in separate analysis, and the data were expressed as either mean or median number of parenchymal, bronchial, and vascular ACE2+ cells/alveolar, bronchial, and vascular tissue area, respectively. Also, the number of ACE2+ MUC5AC+ cells was counted as percentage of total MUC5AC+ cells within the bronchial epithelium.

### ACE2 and TMPRSS2 real time PCR

RNA was extracted from fresh frozen lungs collected at the University of Arizona. Total RNA was isolated from homogenates of lung frozen tissues collected at the University of Arizona using the RNeasy^®^ Mini Kit (Qiagen Inc., Valencia, CA, USA). Purified total RNA was treated with DNase (Invitrogen) according to the manufacturer’s instructions. Reverse transcription was done according to the manufacturer’s instructions using SuperScript™ II RNase H^−^ Reverse Transcriptase (Invitrogen). The primers for the PCR reactions were designed to obtain specific PCR products of similar size for the ORF of each message: ACE2: 5′-TCCATTGGTCTTCTGTCACCCG-3′(Fw.) and 5′-AGACCATCCACCTCCACTTCTC(Rev.)-3′; and TMPRSS2: 5′-CCTCTAACTGGTGTGATGGCGT-3′(Fw.) and 5′-TGCCAGGACTTCCTCTGAGATG-3′(Rev.). Real-time RT-PCR reactions were conducted using iQ™ SYBR^®^ Green Supermix (BIORAD) with the buffers provided at: 95 °C, 10 min, 1 cycle; 94 °C, 10 s; 60 °C, 30 s and 72 °C, 30 s, 40 cycles; with a melt curve over a temperature range starting at 55 °C and ending at 95 °C in a MyiQ™ Cycler (BIORAD). qPCR Calculation for relative quantification was performed using the Delta-delta Ct Method. Human Tubulin was used as an internal housekeeping gene to normalize amplified Ct values for ACE2 and TMPRSS2 for each patient sample (5′- CTTCGGCCAGATCTTCAGAC-3′(Fw.) and 5′- AGAGAGTGGGTCAGCTGGAA-3(Rev.). Briefly, mean Ct values for Tubulin were subtracted from mean Ct for ACE2 and TMPRSS2 Ct values assuming a twofold amplification within each cycle. All samples were run as technical replicates. In a small subset of subjects, we also tested a non-functional isoform of ACE2 reported elsewhere [28].

### Human ACE2 ELISA

Human ACE2 (hACE2) ELISA was performed on lung homogenates of lung frozen tissues collected at the University of Arizona using the Human Angiotensin I Converting Enzyme 2 ELISA Kit (Reddot Biotech Inc., Kelowna, BC V1W 4V3, Canada). Reagents, samples and standards were prepared according to manufacturer’s guidelines. Briefly, 100 µL standard or sample was added to each well and incubated 2 h at 37 °C. This was followed by the addition of 100 µL prepared Detection Reagent A and B, incubate 1 h at 37 °C, in succession. All wells were washed 5 times and 90 µL Substrate Solution was added. Color development occurred optimal at 18 min at 37 °C. Stop Solution was added and the plate was read at 450 nm immediately. ACE2 levels are reported in pg ACE2/µg Total Protein.

### Animal studies

All the murine studies were approved by the Brigham and Women’s Hospital (BWH) IACUC Committee, and performed in compliance with the BWH IACUC and ARRIVE guidelines.

C57BL/6 WT mice were exposed to air or CS for up to 6 months [29, 30]. Adult WT mice were exposed to air or mixed mainstream and sidestream cigarette smoke from 3R4F Kentucky Research cigarettes for 2 h·day−1 on 6 days·week−1 in Teague TE 10z chambers (Teague Enterprises, Woodland, CA, USA) for 1–6 months.

### ACE2 immunofluorescence in murine bronchial and alveolar epithelial cells

To identify ACE2 proteins in murine cells within the alveolar tissue and bronchial epithelial tissue, lung sections were triple immunostained with: (1) mouse anti-ACE2 IgG (1:50 for 2 h at 37 °C, Santa-Cruz biotechnology, Dallas, TX, sc-390851) followed by Alexa-488 IgG (1:100 for 1 h at 37 °C, Fisher Scientific, Hampton, NH, A11001); and (2) rat anti CD326 (EPCAM, 1:50 for 2 h at 4 °C, Biolegend, San Diego, CA, 118201) followed by to Alexa-647 IgG (1:100 for 1 h at 37 °C, Fisher Scientific, Hampton, NH, A21247) and 3) rabbit anti-MUC5AC IgG (1:50 for 2 h at 4 °C, Bioss Antibodies, Woburn, MA, bs7166R) followed by Alexa-568 IgG (1:100 for 1 h at 37 °C, Fisher Scientific, Hampton, NH, A11036). EPCAM was used to identify alveolar and bronchial epithelial cells, and MUC5AC was used to identify goblet cells within the bronchial wall. Lung sections were also immuno-stained with appropriate isotype-matched non-immune control antibodies.

For each sample, 20 randomly-selected high-magnification fields were evaluated using an Olympus epi-fluorescence microscope (Olympus Global Corporation). The number of parenchymal, vascular, and bronchial ACE2+ cells were counted in separate analysis, and the data were expressed as either mean or median number of parenchymal, bronchial, and vascular ACE2+ cells/mm^2^ of alveolar, bronchial, or vascular issue area, respectively. Also, the number of ACE2+ MUC5AC+ cells was counted as percentage of total MUC5AC+ cells within the bronchial epithelium. Tissue area was measured using MetaMorph software (Molecular Devices, Sunnyvale, CA).

### Murine ACE2 ELISA

M-ACE2 ELISA was performed on lung homogenates of lung frozen tissues collected at the Brigham and Women’s Hospital using the Murine Angiotensin I Converting Enzyme 2 ELISA Kit (MyBiosource, San Diego, CA, MBS824565). Reagents, samples and standards were prepared according to manufacturer guidelines. ACE2 levels were normalized for mg of Total Protein.

### In vitro SARS-CoV-2 infections

#### Cigarette smoke extract (CSE)

CSE was made fresh each experiment immediately prior to use following previously validated methods [21, 31]. The cigarettes used were University of Kentucky *3R4F reference* cigarettes (Generous gift from Lingxiang Zhu). Briefly, one entire cigarette was absorbed into 10 mls of BEGM™ in a 75 cm^2^ tissue culture flask. Smoke was pulled up at 50 cc’s at a time and directly injected into the flask, which was immediately re-capped. This was repeated to a total of 250–300 cc's of smoke/10 mls BEGM™ depending on burn rate. The smoke was allowed to completely absorb into the media.

### Calu-3 cell culture

Human lung adenocarcinoma cells derived from metastatic site (ATCC^®^ HTB-55), a line permissive to SARS-CoV-2 infection and replication [32, 33], were purchased from American Type Culture Collection (Manassas, VA) and maintained according to the manufacturer’s instructions. Briefly, cells were initially plated at 30,000 cells/cm^2^ in a T-75 flask and maintained in DMEM high Glucose (Gibco™ 10569-010) supplemented with 10% FBS GenClone™ FetalPURE™ (Cat #: 25-525H). Media changes were made every other day until treatments. To investigate the effects of CS exposure on SARS-CoV-2 infection we performed a 72 h in vitro infection of Calu-3 lung adenocarcinoma cells. Cells were sham- or CSE-treated for 24 h before infection with SARS-CoV-2 isolate (USA-WA1/2020, 2 h viral infection at 3 × 10^e6^ pfu/mL, in normal media, then remove inoculum). Every 24 h Cells were fixed for IF staining, or lysates/supernatants were obtained from the cells for WB of the viral nucleocapsid (N) protein (Novusbio NB100-56683, 1:500 dilution in 1% milk in TBST) and ACE2 (Novusbio NBP1-76614, 1:500 dilution in 1% milk in TBST). IF staining for dsRNA intermediates which arise during viral RNA (vRNA) replication, was performed with dsRNA-specific J2 mAb [32, 34, 35] (Scicons #10010500). Briefly, cells were fixed in PBS with 4% PFA and 4% sucrose for 30 min, permeabilized with 0.2% Triton X-100/PBS for 10 min and blocked with 5% BSA/PBS for 1 h, prior to staining with J2 mAb [32, 34, 35] at 1:1000 dilution at 4C for 1 h. Cells were then washed 3× with 0.1% Tween-20/PBS (PBST) and incubated in secondary goat-anti-mouse Alexa Fluor 546 1:1,000 (Thermo #A11003) for 2 h at room temperature in the dark. Plates were washed 3× with PBST and incubated with DAPI for 30 min at room temperature in the dark. Plates were then imaged with a Nikon Eclipse TI2 automated microscopy system using a 20× objective. Six frames per well were imaged and sum dsRNA fluorescence intensity, normalized to cell count by DAPI, was measured by Nikon Elements imaging software.

### Primary human bronchial epithelial cell culture for air–liquid interface (ALI)

Human bronchial epithelial cell culture kit (Catalog #: CC-2540S) was purchased from Lonza Bioscience (Walkersville, MD). Cells were re-initiated, seeded and maintained according to the company’s protocols. Briefly, cells were initially plated for expansion into in a 75 cm^2^ tissue culture flask at 3500 cells/cm^2^ and maintained in defined serum free Bronchial Epithelial Growth Medium supplemented with corresponding BulletKit™ (Lonza #: CC-3170). After expansion, cells were seeded on rat-tail collagen, 30.0 µg/ml (Corning-Costar, Cambridge MA #354236), coated trans-well plates (Corning-Costar #354236) at 50,000 cells/well and allowed to reach confluence. After confluence was achieved cells were “air lifted” (removal of all media,allowing direct air exposure) and the basal chamber media was changed to B-ALITM Differentiation Media (Lonza Catalog #00193517) supplemented with B-ALITM SingleQuots™ (Lonza Catalog #00193515). Media changes were made to basal chamber daily until treatments.

When confluent, air-exposed ALI-HBECs were washed once with 150 µl PBS on the apical side of the transwell insert to clear mucus. Two hours later, the plates/cells were exposed to freshly generated CSE (see above). Cell exposure was conducted in a modified hypoxic chamber for 5 min to either cigarette smoke at a concentration of 186.7 mg/m^3^ (TSP) or to filtered ambient (“sham”) air. Immediately after, residual smoke (or sham air) was removed by ventilation with ambient air for 5 min and cells were subsequently placed back in the incubator overnight. At 24 h after cigarette smoke exposure, ALI-HBEC were washed apically with PBS and ready for SARS-CoV-2 infection.

### Gamma H2AX expression in HBECs after CSE exposure

To confirm thst CSE was causing oxidative stress on the HBECs, identify gamma H2AX expression, primary HBEC’s were plated on rat tail collagen coated glass coverslips, 30.0 µg/ml, (Corning-Costar, Cambridge MA #354236) at 50% density and allowed to adhere overnight. After either smoke or sham air treatment, cells were immunostained with gamma H2AX [p Ser139] Antibody (Novus Biologicals LLC, Centennial, CO # NB100-384). Briefly, cells were fixed with 4% formaldehyde for 15 min, permeabilize with 0.1% TX-100/PBS for 15 min and blocked with 5% normal goat serum/PBS or 1% BSA/PBS for 1 h. Rabbit anti-human gamma H2AX [p Ser139] Antibody was diluted at 1:250 in blocking solution and cells were allowed to incubate overnight at 4 °C. Following washes, the cells were incubated with goat anti-Rabbit IgG antibody conjugated with Alexa Fluor 488 (Thermo Fisher Scientific, San Diego, CA # A11034) at a dilution of 1:200 in blocking solution for 1 h at room temperature. Post wash, coverslips were dipped into dHO to remove residual salts of the wash buffer and mounted with ProLong™ Gold Antifade Mountant with DAPI (Thermo Fisher Scientific, San Diego, CA #P36935). A secondary only slide was used to evaluate any background staining.

### HBEC SARS-CoV-2 infections

Transwell inserts were setup in fresh 12 well plates with 1 ml basolateral media. SARS-CoV-2 inoculum (MOI of 0.05 PFU/cell) or media alone were added to the apical chambers and incubated for 1 h at 37C. Cells were then washed with PBS to remove all unbound virus, then cells were put back into the incubator and left the top chamber empty for ALI culture over time course. For RT-qPCR assay, total RNA was harvested in with 200ul trizol for RNA extraction using the Direct-Zol RNA mini prep (Zymo research, Cat No. R2050) kit, according to the manufacturer’s instructions. The purified RNA was treated with Turbo DNA-free kit (Invitrogen AM1907) to remove all genomic DNA in the samples. The SARS-CoV-2 nucleocapsid gene was quantified using 2019-nCoV RUO Kit (IDT 10006713) which contain N2 primer sets (Forward Primer: TTA CAA ACA TTG GCC GCA AA, Reverse Primer: GCG CGA CAT TCC GAA GAA) and TaqPath™ 1-Step RT-qPCR method (Thermo fisher A15299), on a QuantStudio6 Flex Real-Time PCR System (ThermoFisher Scientific). Delta-delta-cycle threshold (ΔΔCt) was determined relative to sham treated samples. Viral RNA levels were normalized to housekeeper RNAse P gene (RP, The primer is provided by 2019-nCoV RUO Kit, IDT 1006713, Forward Primer: AGA TTT GGA CCT GCG AGC G, Reverse Primer: GAG CGG CTG TCT CCA CAA GT), and depicted as fold change over vehicle treated samples. Error bars indicate the SEM from n = two or three technical replicates.

For plaque assays, the infected HBEC cells were sampled by addition of 200 uL fresh media to the apical side of the transwell, mixed, and incubated at 10 min at 37C prior to collection. Plaque assay was performed by applying serial dilutions of these samples to confluent Vero-CCL81 cells (ATCC CCL-81-VHG) for 2 h at 37C followed by overlay with complete media containing 1.0% w/v methylcellulose. After 72 h of infection, vero cells were fixed in 10% neutral buffered formalin for 30 min and stained with 0.9% crystal violet for 5 min. Plaques were counted after the staining, and plaque-forming units per ml (PFU/ml) was calculated by “plaque number” × dilution factor × volume factor.

### Statistics

We used one-way analysis of variance tests for continuous variables and Z tests or Chi-square tests for categorical variables. For pairwise comparisons, parametric and nonparametric data were analyzed using two-sided Student’s t tests and Mann–Whitney U tests, respectively. Correlation coefficients were calculated using the Pearson or the Spearman rank method or the Dubin–Watson statistical correlation test for nonlinear data. *P* less than 0.05 was considered statistically significant.

Analyses were performed using SPSS Statistical software.

Since elderly people and male sex are more susceptible to SARS-CoV-2 infection, suggesting that this might due to changes in ACE2 expression with age and sex, distributions for ACE2+ cells in bronchial and alveolar epithelial cells were tested for normality and a log base 10 transformation applied when needed to achieve approximately normal distributions. Multiple linear regression was used to examine the relation of ACE2 measures with patient groups, adjusting for age and sex. A linear combination of estimated coefficients was used to compare COPD and SC groups. Since, in Cohort 2, the NS were significantly younger than SC and COPD, the ACE2 and TMPRSS2 levels were adjusted for age using linear regression models.

## Results

See Additional file 1 and Tables 1 and 2 for additional details on the study subjects. Two separate human cohorts of COPD patients [36], smokers without COPD (“smokers”) and never-smoker controls (NS) were studied.

**Table 1:** Cohort 1. University of Ferrara-COPD and controls

	N	NS	Smokers	COPD	*P*
Total participants (N)	33	7	16	10	
Age	33	71 ± 5	71 ± 9	73 ± 4	NS
Gender (M/F)	33	5/2	9/7	8/2	NS
Smoking history (pack years)	33	0	29 ± 11*	34 ± 11*	*P* < 0.001
Smoking habit (current/former smoker)	33	0	5/11	2/8	NS
Spirometry	33	7	16	10	
FEV1 (% predicted)	33	99 ± 10	104 ± 18	66 ± 10*^^^	*P* < 0.001
FEV1/FVC	33	0.78 ± 0.07	0.81 ± 0.10	0.61 ± 0.08*^^^	*P* < 0.001
Comorbidities	33	7	16	10	
Hypertension N (%)	33	3 (43%)	9 (56%)	6 (60%)	NS
Other cardiovascular diseases N (%)	33	1 (14%)	2 (13%)	2 (20%)	NS
Diabetes mellitus N (%)	33	0	1 (6%)	1 (10%)	NS
Medications	33	7	16	10	
Inhaled corticosteroids N (%)	33	0	0	2 (20%)	NS
LABA/SABA/LAMA N (%)	33	0	0	10 (100%)*^§^	*P* < 0.001
Oral corticosteroids N (%)	33	0	0	0	NS
ACEi/ARB N (%)	33	2 (29%)	4 (25%)	3 (30%)	NS
Ca Antagonists/B-blockers N (%)	33	4 (57%)	9 (56%)	5 (50%)	NS

Data are mean ± SD, unless specified

*NS* never-smokers

*P < 0.001 compared to NS; ^^^*P* < 0.001 compared to Smokers; ^§^*P* < 0.05 compared to NS

**Table 2:** Cohort 2: University of Arizona and Arizona Donor Network: COPD patients and controls

	N	NS	Smokers	COPD	*P*
Total participants (N)	47	10	16	21	
Age	47	56 ± 18*^a^	67 ± 6	66 ± 9	*P* < 0.01^a^
Gender (M/F)	47	7/3	7/9	12/9	NS
Smoking history (pack years)	47	0	33 ± 15***	38 ± 21***	*P* < 0.001
Smoking habit (current/former smoker)	47	0	6/10	5/16	NS
Spirometry	43	7	16	20	
FEV1 (% predicted)	43	77 ± 16	88 ± 21	40 ± 24***^^^	*P* < 0.001
FEV1/FVC	43	82 ± 7	80 ± 9	41 ± 18***^^^	*P* < 0.001
Comorbidities	47	10	16	21	
Hypertension N (%)	47	5 (50%)	9 (56%)	11 (52%)	NS
Other cardiovascluar diseases N (%)	47	1 (10%)	5 (31%)	5 (24%)	NS
Diabetes mellitus N (%)	47	2 (20%)	2 (13%)	4 (19%)	NS
Medications	45	8	16	21	
Inhaled corticosteroids N (%)	45	0	4 (25%)	10 (48%)^§^	*P* < 0.05
LABA/SABA/LAMA N (%)	45	0	6 (38%) §	17 (81%)***^#^	*P* < 0.01
Oral corticosteroids N (%)	45	1 (13%)	0	3 (14%)	NS
ACEi/ARB N (%)	45	3 (38%)	4 (44%)	9 (43%)	NS
Ca Antagonists/B-blockers N (%)	45	2 (25%)	6 (38%)	6 (29%)	NS
Diuretics N (%)	45	2 (25%)	5 (31%)	2 (10%)	NS

Data are mean ± SD, unless specified

*NS* never-smokers

*P < 0.01 versus Smokers and COPD patients; ^§^*P* < 0.05 compared to NS; °*P* < 0.001 compared to smokers and NS; ***P < 0.001 compared to NS; ^#^*P* < 0.01 compared to NS. ^^^*P* < 0.001 compared to smokers

^a^Due to the significant difference in age between NS versus COPD and Smokers, ACE2 levels were corrected for age using linear regression models

**Fig. 1:**
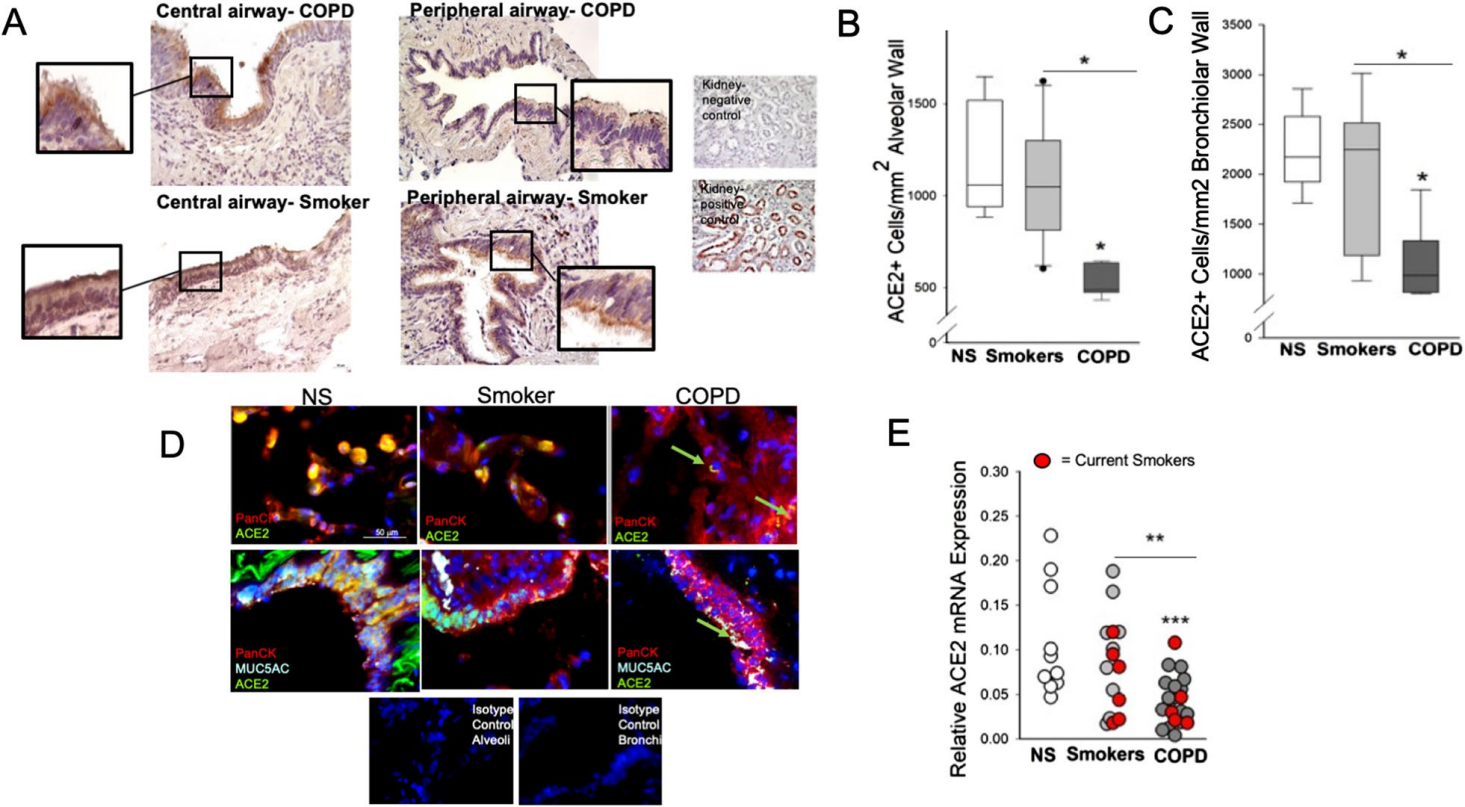
ACE2 expression in bronchial and alveolar epithelium from COPD patients, smoker and never-smoker (NS) controls. The number of ACE2+ cells in the central airway bronchial epithelium was similar between patients with chronic obstructive pulmonary disease (COPD), smokers without COPD and NS controls. In **A**, representative IHC for ACE2 images of central airways of a COPD patient (upper panel) and a smoker without COPD. The insets show details of the ciliated bronchial epithelium. The number of ACE2+ cells in the alveolar epithelium (**B**) and peripheral airway epithelium (**C**), normalized for length of the alveolar wall or basement membrane, respectively, was lower in patients with COPD versus smokers without COPD and NS controls. In **D**, triple immunofluorescence representative images of alveolar (upper panels) and bronchiolar epithelium (lower panels) from a COPD patient, a smoker without COPD, and a never-smoker (NS) where ACE2 staining is identified by green fluorochrome, the epithelium is identified by red fluorochrome, and the color yellow is obtained by merging the two fluorochromes. In **E**, the levels of ACE2 mRNA from peripheral lung samples were decreased between patients with chronic obstructive pulmonary disease (COPD) versus both smoker without COPD and NS controls. The red circles indicate the current smokers among the smoker controls and COPD patients

In Cohort 1, ACE2 expression was mainly observed on goblet cells and on the apical surface of bronchial epithelial cells. No difference was found in ACE2 expression in COPD central airways (5.3% ± 2.9) versus smokers without COPD and NS controls (4.7% ± 2.9 and 7.2% ± 3.5, Fig. 1A and Additional file 1: Fig. S1A). In contrast, ACE2 expression was lower in peripheral airways in COPD patients (mean + SEM: 1.5% ± 0.7) versus both smokers without COPD and NS controls (6.1% ± 1.7; 6.6% ± 2, *P* < 0.05 and *P* = 0.01, respectively, Fig. 1B and Additional file 1: Fig. S1B). Similarly, in Cohort 2 we found both alveolar (mainly type II cells, ATII, Fig. 1B, D) and bronchiolar (mainly club cells and goblet cells, Fig. 1C, D) ACE2 protein expression levels, and ACE2 mRNA pulmonary levels (Fig. 1E) to be lower in COPD patients versus both smokers without COPD and NS controls. Same results were obtained when we measured the mRNA expression of the non-functional isoform of ACE2, that we found lower in COPD versus. NS lungs (Additional file 1: Fig. S2). We did not observe any difference in ACE2 expression in bronchi or alveoli between current versus former smokers, whether they had COPD or not (Fig. 1E, red circles), nor between COPD GOLD stages (not shown). After adjusting for age and sex, ACE2 mRNA levels were all still significantly lower (*P* < 0.05) in COPD lung versus smoker and NS controls. The ACE2 mRNA expression significantly correlated with its protein levels measured by ELISA (*P* = 0.01 and r = 0.71, Additional file 1: Fig. S3) across all the analyzed subjects. At variance with ACE2, TMPRSS2 mRNA levels were not different among groups (*P* = 0.9 for all comparisons). No differences in ACE2 protein levels were observed in vessels between any of the groups.

**Fig. 2:**
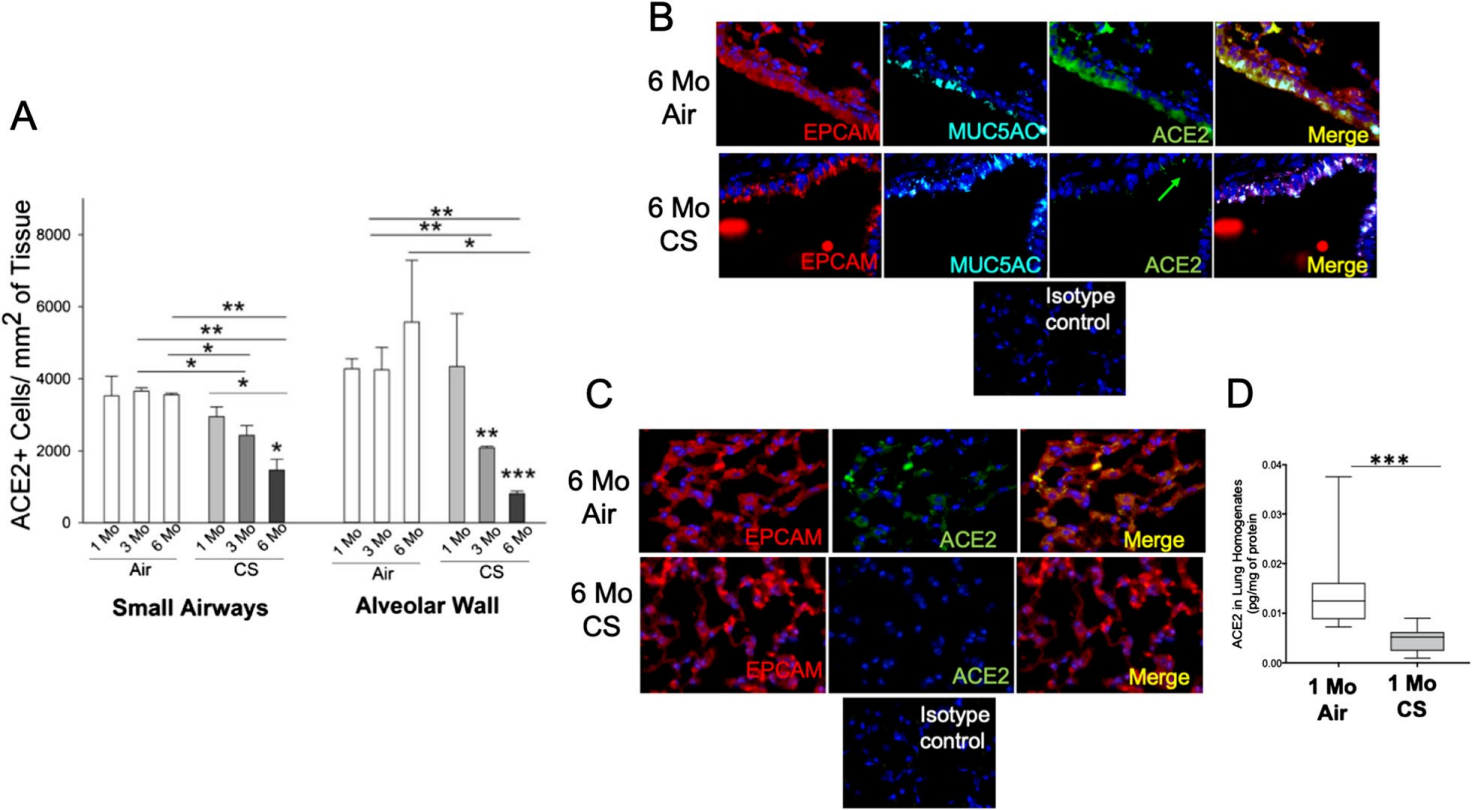
ACE2 expression in bronchial and alveolar epithelium from mice exposed to room air and acutely or chronically to cigarette smoke (CS). WT C57BL6 mice were exposed to air or cigarette smoke (CS) for up to 6 months (n = 3/4 group). In **A**, the number of ACE2+ cells in the bronchial epithelium were decreased in 6-month CS-exposed mice versus 1-, 3-, and 6- month air-exposed mice. The number of ACE2+ cells in the bronchial epithelium was decreased in 3-month CS-exposed mice versus 3- and 6-month air-exposed mice. The number of ACE2+ cells in the bronchial epithelium was decreased in 6-month CS-exposed mice versus 1-month CS-exposed mice. Also, the number of ACE2+ cells in the alveolar epithelium was decreased in 6- and 3-month CS-exposed mice versus 1- and 6- month air-exposed mice. The number of ACE2+ cells in the alveolar epithelium was decreased in 6-month CS-exposed mice versus 1-month CS-exposed mice. In **B**, representative images of air-exposed (upper 3 panels) and CS-exposed (lower 3 panels) murine small airways where bronchial epithelial cells are identified by staining with a red fluorophore, mucin-producing cells by a cyan fluorochrome, and ACE2+ cells by a green fluorophore. In **C**, representative images of air-exposed (upper three panels) and CS-exposed (lower three panels) murine alveolar cells where the epithelial cells are identified by staining with a red fluorophore, and ACE2+ cells by a green fluorophore. The staining isotype control for each staining is also shown. In **D**, the ACE2 protein levels measured by ELISA in total lung homogenates were decreased in mice exposed to CS for one month versus air (n = 10–15/group). * = *P* < 0.05; ** = *P* < 0.01; *** = *P* < 0.001. *CS* cigarette smoke

In mice, we found that the longer the CS exposure, the lower the levels of ACE2 staining in both bronchial (Fig. 2A, B) and alveolar (Fig. 2A, C) epithelial cells. As expected, the mice exposed to CS for 6 months had developed a COPD-like phenotype with airspace enlargement and small airway remodeling [30, 37], and had significantly higher MUC5AC + bronchial cell numbers versus air-exposed mice. Similarly, CS-exposed mice had a lower % ACE2+ MUC5AC+/total MUC5AC+ cells in the bronchial epithelium versus air-exposed mice (*P* < 0.05 for all comparisons, not shown). ELISA-measured pulmonary ACE-2 protein levels were significantly lower in 1-month CS- versus air-exposed mice (*P* < 0.001, Fig. 2D).

In vitro CS exposure increased the levels of gamma H2AX in both Calu3 and HBECs, confirming that the CS was inducing oxidative stress in the cells (Additional file 1: Fig. S4). The same effect was not observed in sham-treated cells. Also, viability assays confirmed that the CS concentrations used for the exposures prior to SARS-CoV-2 infection were not lethal for the cells.

**Fig. 3:**
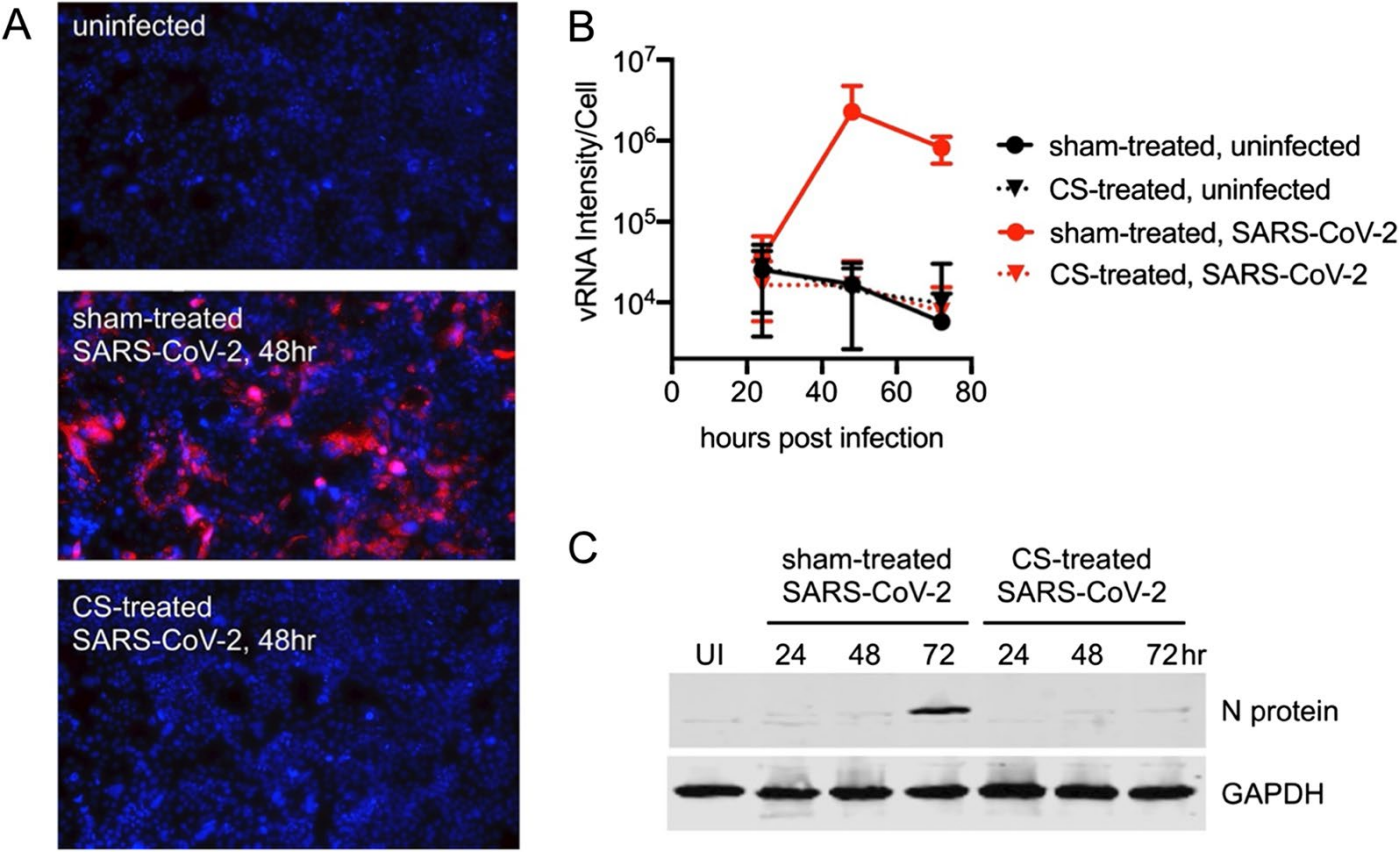
Cigarette Smoke (CS) extract blocks in vitro SARS-CoV-2 replication in Calu-3 cells. To investigate the effects of cigarette smoke (CS) exposure on SARS-CoV-2 infection, we performed a 72 h in vitro infection of Calu-3 cells, a line permissive to SARS-CoV2 infection and replication. Cells were sham- or CSE-treated for 24 h. Supernatants (SN) and cytoplasmic lysates were obtained from a cell aliquot to measure ACE2 levels by ELISA. Then, cells were infected with SARS-CoV-2 (2 h viral infection in normal media, then remove inoculum). Every 24 h cells were fixed for IF staining of infection, and cell lysates were harvested for SDS-PAGE and WB of viral nucleocapsid (N) protein. dsRNA intermediates arise during the replication of viral RNA (vRNA), and IF staining with dsRNA-specific J2 monoclonal Ab is a good marker for SARS-CoV-2 replication. A Nikon Ti2 automated microscopy was used to quantitatively measure infection, as seen by dsRNA signal. Whereas replication of vRNA peaked at 48 h (**A, B**) in sham-treated cells, CSE-treatment abrogated infection to levels below the limit of detection. Similar results were seen with WB for viral N protein, showing peak viral protein synthesis at 72 h (**C**). In **C**, immunoblots show two bands used for densitometry and separated with a horizontal white line, one for the N protein, and one for GADPH. The two bands were cropped from original gels that are available in a Supplementary repository. ACE2 protein levels were undetectable in the SN, but were unchanged in CSE-treated versus sham cell lysates (not shown). In summary, CSE-pre-exposure increased ACE2 levels but potently abrogated SARS-CoV-2 replication in this in vitro model. The figure is representative of three independent experiments

**Fig. 4:**
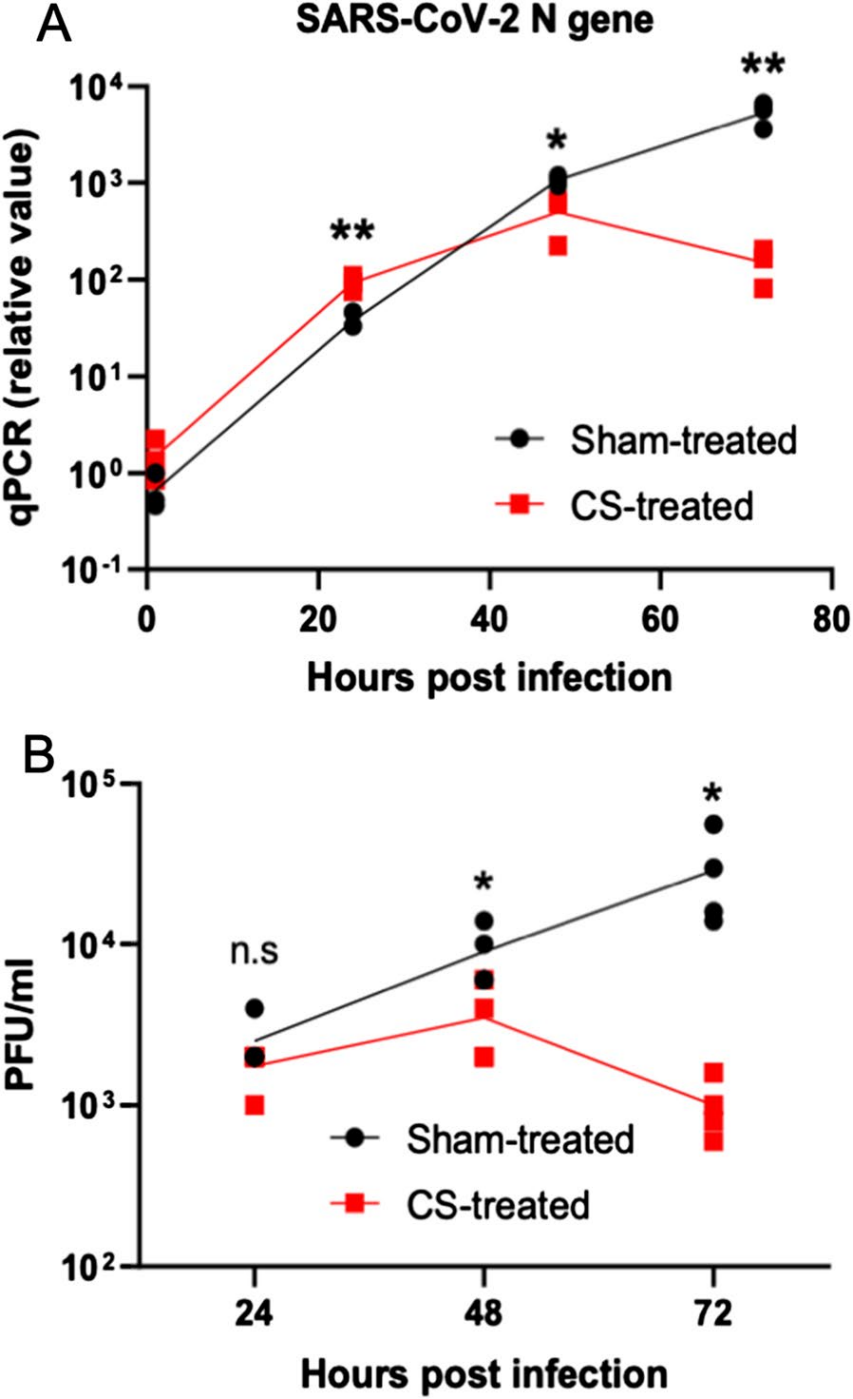
Acute CS treatment blocks in vitro SARS-CoV-2 replication in differentiated primary airway epithelium. HBECs, fully differentiated into airway epithelium by culture at air–liquid interface, were subjected to acute sham- or CS-exposure prior to SARS-CoV-2 infection. Cells were infected via apical inoculation with SARS-CoV-2 at 0.05 PFU/cell MOI for 1 h at 37C, washed to remove unbound virus, and infections were incubated for 24 h, 48 h, and 72 h. **A** Samples are collected for RNA purification and RT-qPCR to detect the level of SARS-CoV-2 nucleocapsid gene in infected cells by following the procedure described in method session. The SARS-CoV-2 N gene level is lower in 48 h and 72 h post-infected CS-treated cells compare to Sham-treated cells (n = 3 technique replicates). * = *P* < 0.05. ** = *P* < 0.01. *CS* cigarette smoke. **B** The infected HBEC cells were sampled by addition of 200 uL fresh media to the apical side of the transwell to collect all the progeny virus that is released from the infected cells. The amount of the progeny viruses was tittered by plaque assay as described in method session. CS-treated cells generated less SARS-CoV-2 progeny compared to Sham-treated cells after 48 and 72 h of infection (n = 4). * = *P* < 0.05. *CS* cigarette smoke

In vitro SARS-CoV-2 infection of Calu3 cells was readily observed by dsRNA staining (Fig. 3). Whereas replication of vRNA peaked at 48 h in sham-treated cells, CS-exposure abrogated infection to levels below the detection limit. Similar results were seen with viral N protein expression, showing peak viral protein synthesis at 72 h. ACE2 protein levels were undetectable in the supernatants, whereas they were unchanged in CS-treated versus sham cell lysates (not shown). Similarly, SARS-CoV-2 infection of HBECs resulted in replication of vRNA peaking at 72 h in sham-treated cells, whereas acute CS-exposure decreased the infection starting from 48 h post infection (Fig. 4A). Plaque assay confirmed the CS-dependent inhibition of viral replication, with significantly decreased levels of infectious SARS-CoV-2 PFU at both 48 h and 72 h post infection (Fig. 4B).

## Discussion

The effects of CS and COPD on SARS-CoV-2 infection and disease a still a matter of debate. If on one hand epidemiological studies worldwide have shown a lower than expected prevalence of active smokers and COPD among COVID-19 patients [7–14], smokers who develop COVID-19 are more likely to have worse COVID-19-related outcomes [38, 39]. Herein we show that CS and COPD are associated with lower ACE2 levels in both human and murine cohorts, and CS-pre-exposure potently abrogates SARS-CoV-2 replication in this in vitro model. Our findings in human samples are in line with some recent studies that found ACE-2 levels to be decreased in the lung tissue of smokers with severe COVID-19 pneumonia [40], but are not aligned with some others [18–21, 41]. Zhang et al. [19] assessed the ACE2 gene expression in NS and smokers, finding ACE2 expression to be higher in central airways. Consistently with theirs and others’ findings, we found. While Zhang et al. did not include COPD patients in their analyses, Cai et al. [18] and Smith et al. [41] analyzed several datasets of gene expression of COPD and control small and large airway epithelium samples, concluding that ever-smoking was the main factor associated with high ACE2 levels; however, the effect of COPD was not consistent across datasets. Leung et al. [20] reported higher pulmonary ACE2 protein and mRNA levels in smokers versus NS and COPD versus non-COPD. Additionally, they quantified ACE2 mRNA levels in subsegmental airway brushings, whereas we assessed peripheral lung resections. Leung et al. also showed a diffuse increased ACE2 staining in bronchiolar epithelial cells from COPD versus controls, while we detected apical staining in bronchiolar cells, with diffuse expression only in goblet and club cells. Jacobs et al. [21] reported higher ACE2 protein and mRNA levels from ever smokers without and with COPD versus NS. However, there are several differences in the methods (e.g., primers for different ACE2 splice variants) and study population (higher prevalence of hypertension, treatment with oral corticosteroids, LABA and LAMA, all known to affect ACE2 levels [42, 43], and a much higher percentage of females and current smokers) employed in our study. Nonetheless, the magnitude of ACE2 expression found in the central and peripheral airways from our study subjects is in line with that found in other studies [21, 44–46]. Of note, soluble ACE2 can be released from the epithelial surface into the airway lumen via sheddase cleavage [47, 48], and thus, a dynamic expression of ACE2 in the airways in response to noxious stimuli such as CS and COPD could underlie such variability in the findings.

CS-exposed mice had decreased ACE2+ cells in both alveolar and bronchial epithelial cells versus air-exposed mice, with the lowest number of ACE2+ cells found after six months of CS exposure. A previous study showed a lower ACE2 mRNA expression in the lungs of CS-exposed rats with a COPD-like phenotype compared with that in air-exposed animals [49]. In contrast, in a recent study, higher pulmonary ACE2 levels were found in CS-exposed versus air-exposed mice [41], but the smoke exposures were shorter than the ones used in our study, which might have resulted in less airway remodeling and emphysema. Further, cell subtypes expressing ACE2 in the CS-exposed lungs were not assessed.

Finally, we show for the first time that CS potently blocks SARS-CoV-2 replication in both Calu-3 and primary HBECs in vitro. There is controversy over smoking and SARS-CoV-2 cell line infection models. Acute CS exposure has been shown to reduce the innate immune response and airway basal stem cell proliferation in vitro [50], and to increase ACE2 levels, mACE2 activity, and sACE2 in primary bronchial epithelial cultures [51]. However, these discrepancies are not surprising as there is a wide variation in the differentiation capacity of airway epithelial cells in ALI cultures from different donors especially in response to CS stimulation, which itself can introduce a high grade of variability depending on the methods used to generate it, together with variation in the number of SARS-CoV-2 infected cells in each set of cultures. Therefore, it can be challenging to assess effects across heterogeneous patient samples. Also, the timing of cell infection with SARS-CoV-2 is critical, and the point of point of maximal viral load didn’t necessarily correspond with the biggest changes in cell types. Taken together, these studies, when conflated with features of attributable COVID-19 lung morbidity, indicate that lung ACE2 expression is a suboptimal marker of SARS-CoV-2 susceptibility and COVID-19 expression and morbidity. Additional factors likely play a role in the interaction between smoking, COPD, and SARS-CoV-2 infection. First, whether elevated ACE2 levels are beneficial or harmful during SARS-CoV-2 infection is still unclear. Importantly, a key study published by Imai et al. [52] showed that ACE2 protects mice from severe acute lung injury induced by sepsis. Also, since ACE2 expression declines significantly from the upper airways to the lung, we speculate that the susceptibility to the virus should be mostly correlated to the presence of ACE2 in the nasal mucosa and upper airways [53]. Second, naturally-occurring structural changes in the ACE2 allelic variants could interfere with the intermolecular interactions with SARS‐CoV-2 spike protein and thus the viral entry [54]. Third, nicotine interacts with many components of the renin-angiotensin system in multiple organ systems by downregulating the expression and/or activity of ACE2 and Angiotensin II Receptor [24]. Also, the activation of nicotinic acetylcholine receptors, that are abundant in the bronchial epithelium [55], can lead to enhanced activity of proteinases that cleave the spike protein of SARS-CoV-2 needed for membrane fusion, leading to impaired viral entry into the host cells. Fourth, soluble ACE2 can be released from the epithelial surface into the airways via sheddase cleavage [47, 48]. Soluble ACE2 release is a dynamic process, occurring both constitutively and in response to various stimuli, possibly CS and COPD.

We acknowledge that a major limitation of this study is the lack of insight into the mechanisms by which CS mediates SARS-CoV-2 infection and disease. Nonetheless, we believe that our pilot in vitro findings are both extremely novel and striking and can pave the way to future studies aimed at understanding the effects of CS on SARS-CoV-2 infection. Also, our in vitro findings with acute CS exposure do not recapitulate our in vivo observations of decreased ACE2 levels in response to chronic CS. Last, we acknowledge that other putative SARS-CoV-2 receptors/proteases e.g. cathepsin L, BSG, Adam17 could play a role in SARS-CoV-2 infection, and their analysis is currently ongoing.

## Conclusions

In conclusion, we report in two independent human cohorts, lower protein and mRNA levels of ACE2 in both alveolar and bronchiolar epithelium of COPD patients versus smokers without COPD and NS controls. Consistently, we report that ACE2 levels were reduced in mice exposed chronically to CS versus air-exposed mice. Last, we report that CS pre-exposure potently decreased SARS-CoV-2 replication in this in vitro model. These results point at complex biological interactions between CS, COPD, ACE2, and SARS-CoV-2 entry in the host cells that need to be further explored in future clinical and translational studies.

## Supplementary Information

**Additional file 1: Fig. S1.** No difference was found in ACE2 expression in COPD central airways vs. smoker and NS controls. In contrast, ACE2 expression was lower in peripheral airways in COPD patients vs. both smoker and NS controls. **Fig. S2.** mRNA expression levels of ACE2 in a subset of never smokers and COPD lungs. The primers used were from Blume C. et al. Nat. Genet. 2021. **Fig. S3.** Significant correlation between ACE2 mRNA expression and its protein levels measured by ELISA (P = 0.01 and r = 0.71) across all the analyzed subjects (COPD, smoker and NS controls). **Fig. S4.** In vitro CS exposure increases the levels of gamma H2AX in human bronchial epithelial cells (HBECs), confirming that the CS was inducing oxidative stress in the cells.

## Data Availability

Raw data will be available from the corresponding author on reasonable request.

## Abbreviations

ALI: Air–liquid interface
COPD: Chronic Obstructive Pulmonary Disease
COVID-19: Coronavirus Disease 2019
CS: Cigarette smoke
GOLD: Global Initiative for Obstructive lung Disease
HBECs: Human Bronchial Epithelial Cells
ICS: Inhaled corticosteroid
LABA: Long-acting beta agonist
LAMA: Long-acting muscarinic antagonist
NS: Never smokers
