# Supplementary material for: Paradoxical effects of cigarette smoke and COPD on SARS-CoV-2 infection and disease
Source: BMC Pulm Med. 2021 Aug 23;21:275. doi: 10.1186/s12890-021-01639-8 (PMC8381712; doi:10.1186/s12890-021-01639-8)
Supplement: Supplementary file 1 — Additional file 1: Fig. S1. No difference was found in ACE2 expression in COPD central airways vs. smoker and NS controls. In contrast, ACE2 expression was lower in peripheral airways in COPD patients vs. both smoker and NS controls. Fig. S2. mRNA expression levels of ACE2 in a subset of never smokers and COPD lungs. The primers used were from Blume C. et al. Nat. Genet. 2021. Fig. S3. Significant correlation between ACE2 mRNA expression and its protein levels measured by ELISA (P = 0.01 and r = 0.71) across all the analyzed subjects (COPD, smoker and NS controls). Fig. S4. In vitro CS exposure increases the levels of gamma H2AX in human bronchial epithelial cells (HBECs), confirming that the CS was inducing oxidative stress in the cells. [file 12890_2021_1639_MOESM1_ESM.pptx]

## Slide 1
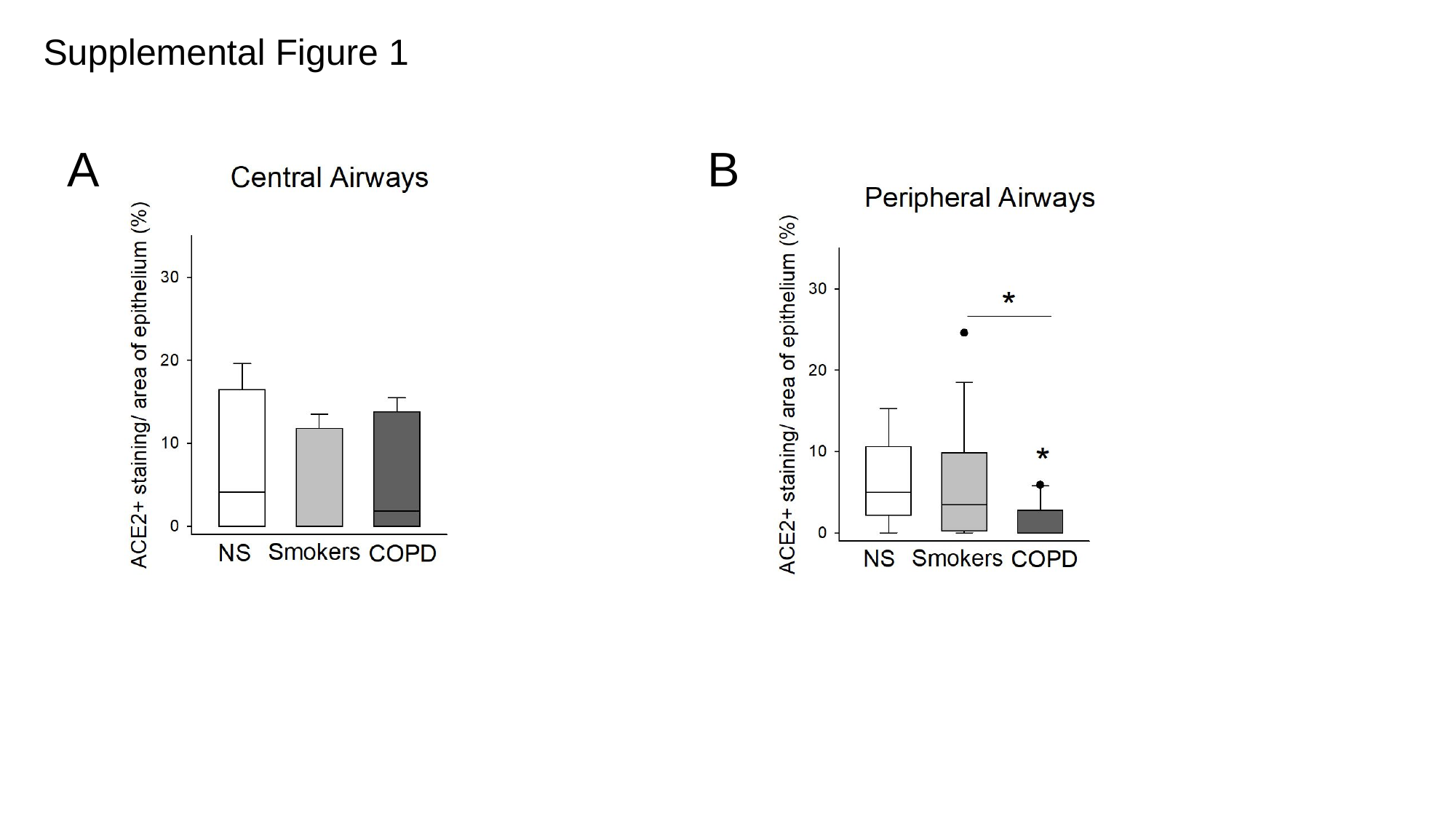

Supplemental Figure 1
A
B

## Slide 2
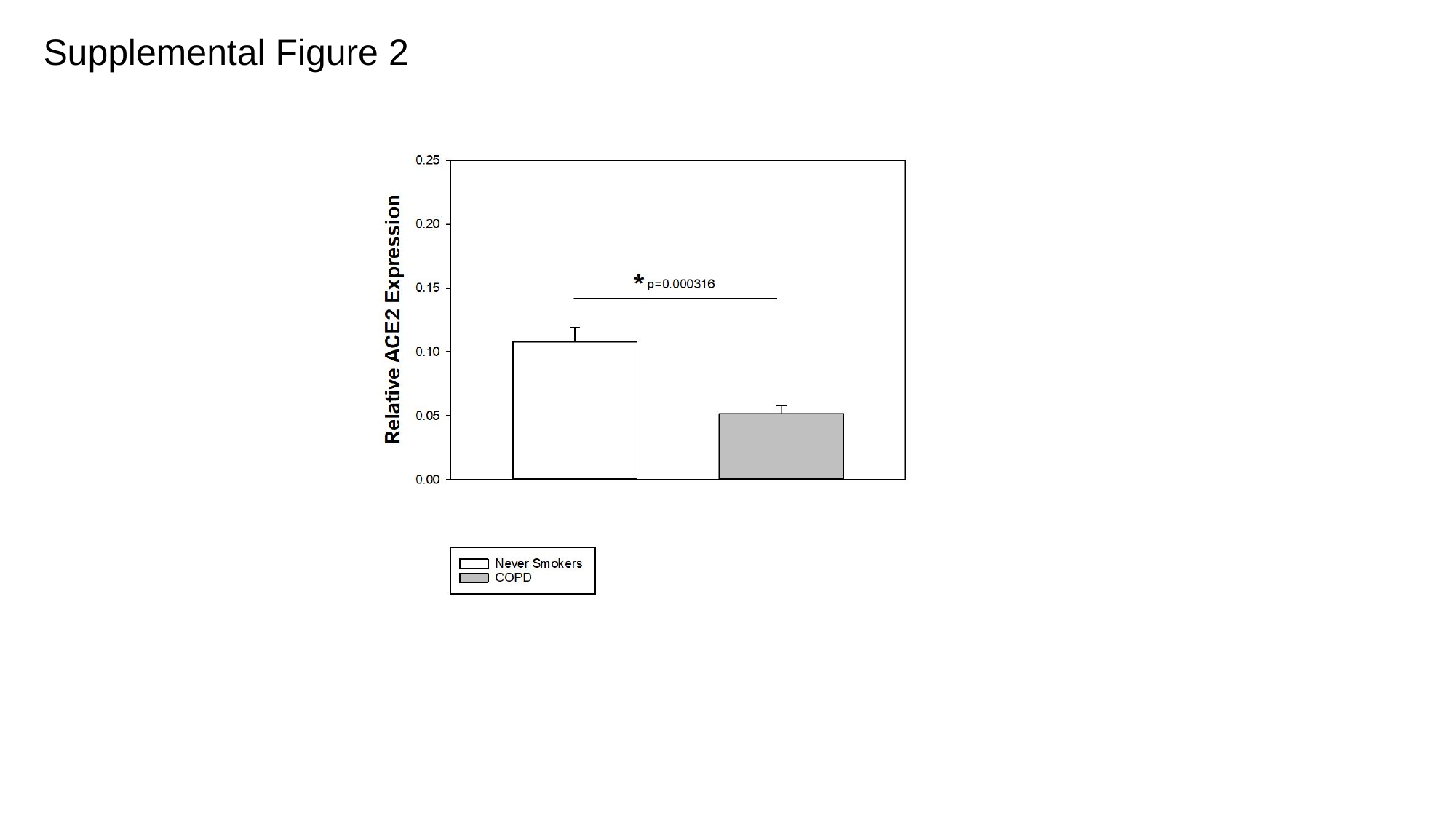

Supplemental Figure 2

## Slide 3
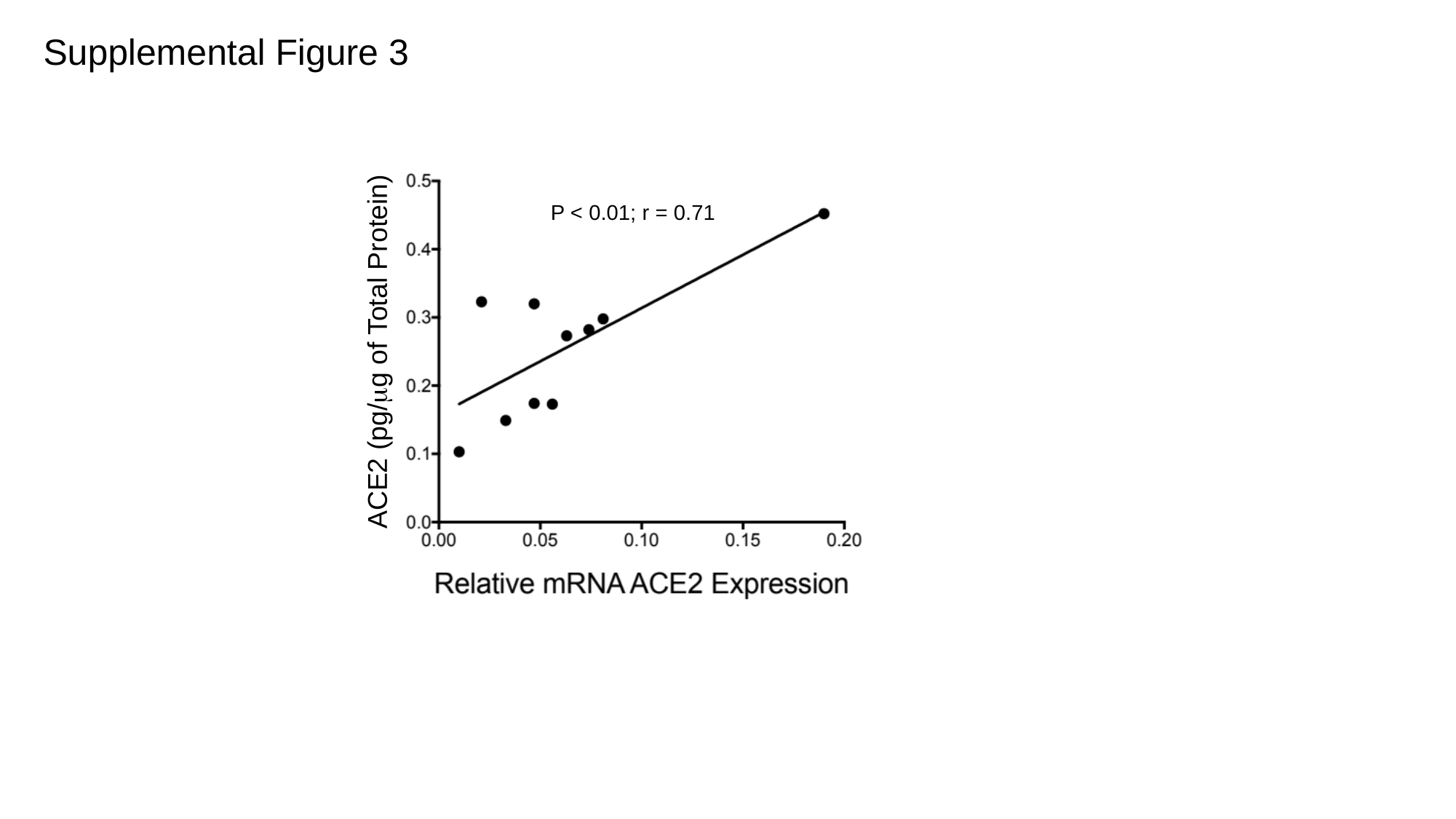

Supplemental Figure 3
ACE2 (pg/mg of Total Protein)
P < 0.01; r = 0.71

## Slide 4
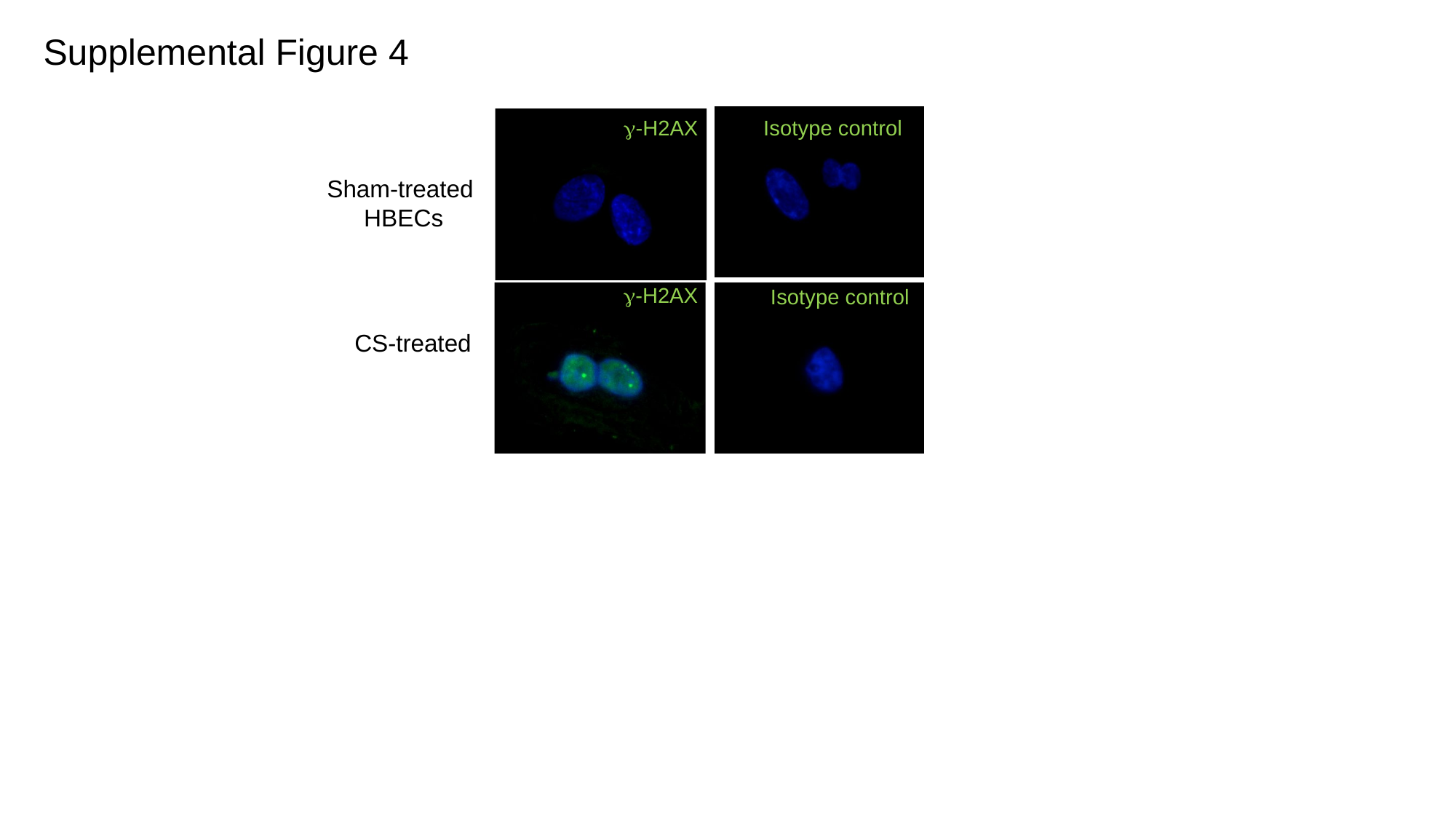

Supplemental Figure 4
Isotype control
g-H2AX
Sham-treated
HBECs
g-H2AX
Isotype control
CS-treated
